# Pulmonary mass‐like lesion caused by *Toxoplasma gondii* in a domestic shorthair cat

**DOI:** 10.1111/jvim.16111

**Published:** 2021-05-04

**Authors:** Myles McKenna, Monica Augusto, Alejandro Suárez‐Bonnet, Ella Fitzgerald

**Affiliations:** ^1^ Section of Veterinary Clinical Sciences University College Dublin Dublin Ireland; ^2^ Department of Pathobiology and Population Sciences Royal Veterinary College Hatfield UK; ^3^ Dublin Ireland

**Keywords:** lung, pneumonia, protozoa, respiratory, toxoplasmosis

## Abstract

A 2‐year‐old male neutered domestic shorthair cat underwent investigations for acute onset of lethargy, hyporexia, and cough. Computed tomography of the thorax identified a large mass‐like lesion in the left cranial lung lobe and bilateral pleural effusion. Thoracotomy and left cranial lung lobectomy were performed. Histopathology of the pulmonary mass was consistent with a localized *Toxoplasma gondii* pneumonia, confirmed by positive polymerase chain reaction on the affected lung lobe. After adjunctive medical management with a 28‐day course of clindamycin (12.5 mg/kg PO q12h), clinical signs resolved and repeat thoracic radiographs documented no abnormalities. The cat remains clinically well 1 year after surgery.

**Abbreviations**
CT: computed tomography
FeLV: feline leukemia virus
FIV: feline immunodeficiency virus
IgG: immunoglobulin G
IgM: immunoglobulin M
RI: reference interval

## 1 CASE DESCRIPTION

A 2‐year‐old male neutered domestic shorthair cat was initially presented to a primary care veterinarian in the United Kingdom for evaluation of a 4‐day history of lethargy, hyporexia, and intermittent cough. The cat had no prior relevant medical history and was on no medications, apart from monthly topical fipronil (Frontline, Boehringer Ingelheim, Ingelheim am Rhein, Germany) for ectoparasite prevention. It had been adopted from a rescue center approximately 4 months before presentation and had regular outdoor access. On physical examination at that time, the cat was bright, alert and responsive. It was tachypneic with a respiratory rate of 44 breaths per minute and a restrictive breathing pattern. Pulmonary parenchymal sounds and heart sounds were decreased ventrally, bilaterally, more notably on the left side. Heart rate and rectal temperature were within normal reference intervals (RIs). No other abnormalities were present on physical examination.

**FIGURE 1:**
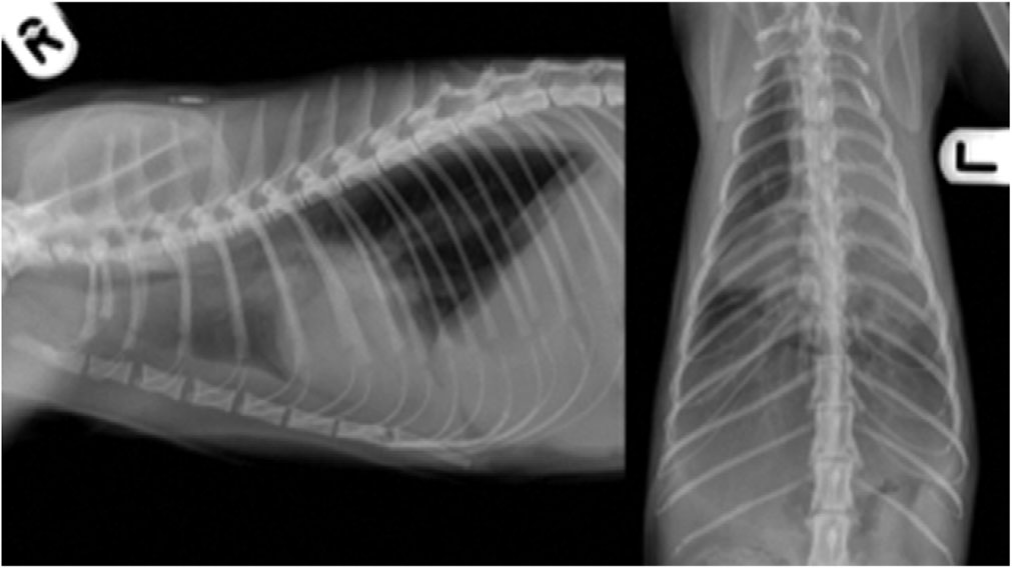
Right lateral and dorsoventral projections of the thorax. There is increased soft tissue opacity in the left cranial and right middle lung lobes. Pleural effusion is present

Thoracic radiographs, reviewed by a Diplomate of the European College of Veterinary Diagnostic Imaging, revealed moderate pleural effusion bilaterally and a pulmonary mass‐like lesion associated with the left cranial lung lobe. There was a second area of increased pulmonary opacity in the right hemithorax associated with the right middle lung lobe (Figure 1). Peripheral capillary oxygen saturation (SpO2) was 98%. Ultrasound‐guided thoracocentesis was performed for diagnostic and therapeutic purposes, and 50 mL of slightly turbid yellow‐orange effusion was drained from the left hemithorax. The effusion was a protein‐rich transudate (total nucleated cell count was 1.4 × 10^9^/L and total protein was 3.0 g/dL) with a population of atypical large mononuclear cells. There was no aerobic or anaerobic bacterial growth. There was a marked leukopenia of 2.1 × 10^9^/L (RI, 7.7‐19 × 10^9^/L), comprised of a marked neutropenia (0.65 × 10^9^/L; RI, 2.5‐12.5 × 10^9^/L) and moderate lymphopenia (0.9 × 10^9^/L; RI, 1.5‐6.5 × 10^9^/L). No white blood cell morphological abnormalities were identified. There was mild hypoproteinemia of 5.1 g/dL (RI, 5.5‐7.8 g/dL) characterized by mild hypoalbuminemia (1.9 g/dL; RI, 2.2‐3.6 g/dL). Serum submitted for feline leukemia virus (FeLV) and feline immunodeficiency virus (FIV) serology (SNAP Combo FeLV Antigen/FIV Antibody Test [bidirectional flow ELISA], Idexx Laboratories) was negative for both agents.

**FIGURE 2:**
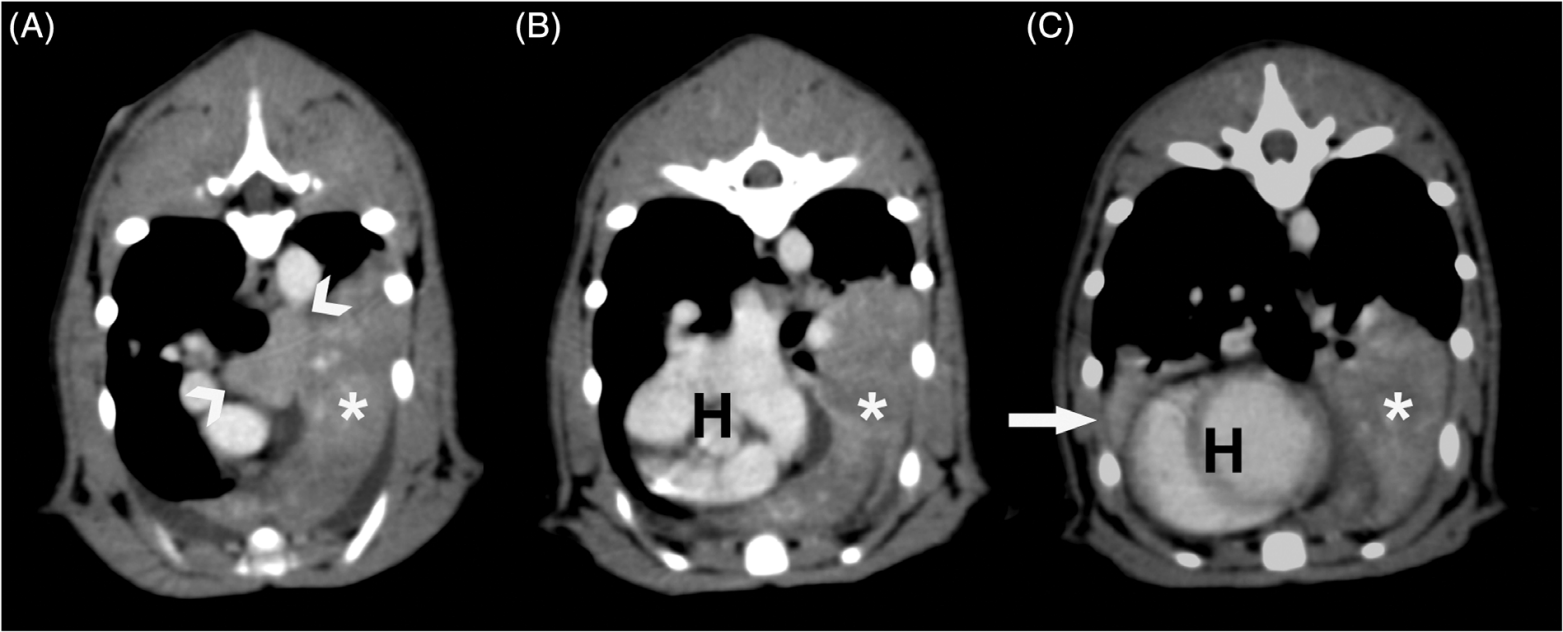
Post contrast CT images of the left cranial lung lobe mass‐like lesion (*), enlarged cranial mediastinal lymph node (arrowheads), and collapsed right middle lung lobe (white arrow). A‐C demonstrate the cranial aspect, middle, and caudal aspect of the mass‐like lesion, respectively. The heart is annotated by H

To further investigate the pulmonary mass‐like lesion seen on thoracic radiographs, a contrast computed tomography (CT) scan of the thorax and abdomen was performed under general anesthesia (320‐slice CT scanner [Canon Medical Systems, Aquilion ONE Genesis Edition, Tochigi, Japan] with power injector [Bayer PLC, Medrad Stellant, Leverkusen, Germany]). Preanesthetic echocardiography revealed a structurally normal heart. There was a soft tissue mass in the left cranial lung. This lesion was causing a slight mass effect with shift of the mediastinum to the right. The mass exhibited uneven enhancement after administration of contrast. Additional findings included cranial mediastinal lymphadenopathy, a moderate volume of pleural fluid and a small volume of pleural gas (Figure 2). The right middle lung lobe was collapsed. No abnormalities were identified in the abdomen. Cytology of ultrasound‐guided fine needle aspirates of the left cranial lung lobe mass‐like lesion revealed marked pyogranulomatous inflammation with protozoal microorganisms, most consistent with *Toxoplasma* spp. tachyzoites. There was evidence of necrosis and previous hemorrhage. *Toxoplasma* serology (indirect immunofluorescence assay [IFA], Biobest Laboratories Ltd, United Kingdom) revealed an immunoglobulin G (IgG) titer >1 : 800 and immunoglobulin M (IgM) titer <1 : 20.

**FIGURE 3:**
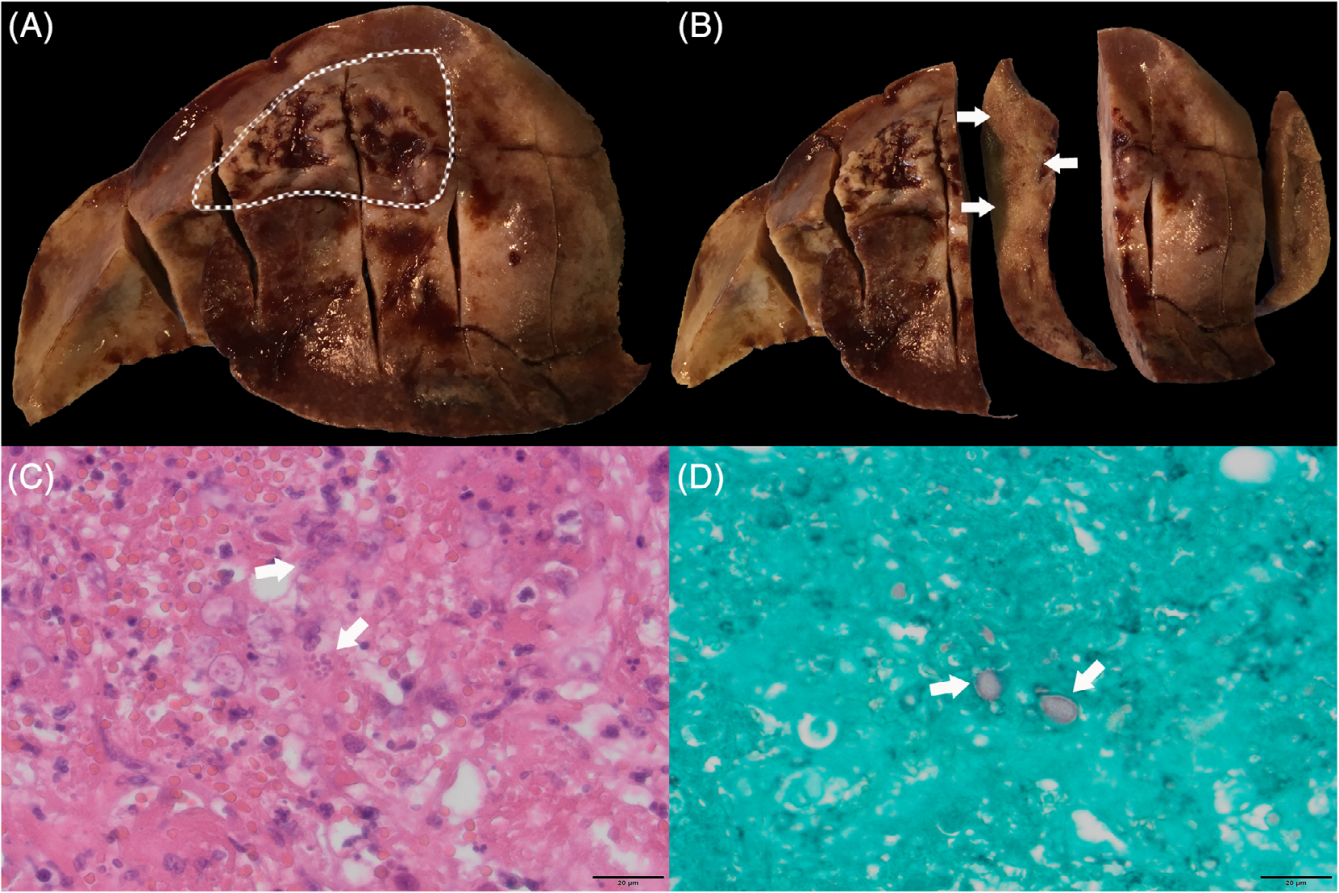
A, Gross image of the left cranial lung lobe after surgical resection. A poorly demarcated nodule, outlined by the dashed white line, is present on the medical aspect of the lung lobe. B, Gross image of the cut surface of the medial aspect of the left cranial lung lobe. Effacement of the normal lung parenchyma with replacement by tan‐brown tissue is noted. C, Photomicrograph at high power (60×) of the *Toxoplasma gondii* lesion. Cysts containing multiple *T gondii* organisms are demonstrated by white arrows. D, Photomicrograph at high power (60×) of the *T gondii* lesion after staining with Gomori Methenamine‐Silver, highlighting the walls of the parasitic cysts (white arrows)

Given the extensive size of the left cranial lung lobe mass‐like lesion, which was assessed to most likely represent a *Toxoplasma* granuloma, and the evidence of necrosis on cytology, it was elected to pursue surgical management rather than solely medical treatment of the toxoplasmosis. A left lateral 5th intercostal thoracotomy and left cranial lung lobectomy were performed. The left cranial lung lobe was removed using a TA30 V3 stapler (Medtronic DST Series TA, Covidien). Grossly, the medial aspect of the left cranial lung lobe was expanded by a 3.2 × 1.4 cm raised, poorly demarcated nodule (Figure 3A). The cut surface exhibited effacement of the normal lung parenchyma and was replaced by a firm, tan‐brown tissue (Figure 3B). Histopathology of the excised left cranial lung lobe revealed severe, multifocal to coalescing, subacute to chronic, lymphohistiocytic, necrotizing bronchointerstitial pneumonia, with occasional intralesional parasitic cysts. Cysts contained multiple 3‐4 × 2 μm, banana‐shaped organisms with distinct eccentric nuclei that were consistent with *Toxoplasma gondii* organisms (Figure 3C). Serial tissue sections from these areas of pneumonia were stained with Gomori Methenamine‐Silver which highlighted the walls of parasitic cysts (Figure 3D). Polymerase chain reaction (PCR) for *T gondii* (*T gondii* RealPCR, Idexx Laboratories, Wetherby, United Kingdom) on the left cranial lung lobe was positive. Aerobic and anaerobic cultures of the affected lung lobe were negative.

Clindamycin (Antirobe, Zoetis, United Kingdom) was administered at a dose of 12.5 mg/kg PO q12h as medical treatment for toxoplasmosis. The cat's respiratory rate and effort normalized after thoracotomy, and it was discharged 4 days postoperatively, with instructions to continue administration of clindamycin for a total of 4 weeks, and to administer buprenorphine (Buprenodale Multidose, Dechra Veterinary Products, Shrewsbury, United Kingdom) 0.02 mg/kg sublingually (PO) every 8 hours for a further 5 days.

On recheck examination 4 weeks later, the cat's owners reported it to have been clinically normal since the time of discharge. Physical examination at this time revealed no abnormalities. Repeated thoracic radiographs revealed no changes, except for the absence of the left cranial lung lobe. Repeat IgG and IgG IgM titers were >1 : 800 and <1 : 20, respectively, and on hematology the previous neutropenia had fully resolved (neutrophil count 4.2 × 10^9^/L; RI, 2.5‐12.5 × 10^9^/L). The cat remains clinically well 1 year after the time of presentation.

## 2 DISCUSSION

This is a case of localized pulmonary toxoplasmosis in a cat. Pulmonary toxoplasmosis is documented previously in cats. However, in other reported cases, the disease presented as part of disseminated toxoplasmosis, with multiple pulmonary lesions or diffuse alveolar or interstitial pulmonary changes, with or without pleural effusion.^1^, ^2^, ^3^ Affected cats have an identifiable cause of systemic immunosuppression that predisposed them to develop clinical toxoplasmosis, such as the administration of chemotherapy,^1^ cyclosporine treatment,^2^, ^4^ or pregnancy.^3^ Conversely, the cat in this report had a single large pulmonary lesion and had no identifiable cause for immunosuppression.

Infection of cats with *T gondii* is common and immunocompetent cats rarely develop clinical disease. The cat in this case report developed clinical toxoplasmosis despite apparently being immunocompetent. The cat had no recent history of receiving immunosuppressive medications. It was screened for FIV and FeLV at presentation, 2 of the most common infectious causes of immunosuppression, and tested negative. Although the SNAP FIV/FeLV Combo Test is highly sensitive for both infectious agents,^5^ the authors acknowledge that false negative results can occur in cases of recent infection with 1 or both of these viruses. In the case of FIV, it is possible that seroconversion could have been identified had serological testing been repeated, whereas in the case of FeLV it is possible to have a false negative result in the first 30 days after infection, before antigenemia develops.^6^ Although the cat in this case was markedly neutropenic at the time of presentation, the neutropenia was transient. The authors believe the neutropenia was therefore more likely a response to systemic inflammation associated with toxoplasmosis, rather than indicative of an underlying immunodeficiency. The possibility that this cat was immunocompetent and developed clinical disease due to a highly pathogenic strain of *T gondii* was considered unlikely as the current scientific literature does not support a relationship between genotype of *T gondii* and clinical outcome or presentation.^7^, ^8^ The cat could have had an immunodeficiency that was not detected.

The cat in this report had IgG titers exceeding 1 : 800 both at the time of presentation and on repeat sampling 4 weeks later, whereas IgM titers were not detected on either occasion. Although most cats exposed to *T gondii* are positive for IgM within 2 weeks of infection, some cats do not have detectable IgM until 4 to 10 weeks after infection, and up to 20% of infected cats never develop detectable IgM.^9^, ^10^ Unfortunately, the IgG titer on both occasions it was measured exceeded the maximum detectable dilution tested by the laboratory. Had further dilutions been available, assessing the dynamics of the IgG titer over time would have been helpful for characterizing the chronicity of the immune response and timeframe of possible exposure. It is possible the cat in this report had a novel *T gondii* infection caused by ingestion of bradyzoites from rodent prey or undercooked meat. An alternative explanation is that this cat's toxoplasmosis was caused by reactivation of a latent infection, triggered by an unidentified immunosuppressive stress.

When a pulmonary mass‐like lesion is documented on thoracic radiography or CT, the primary differential is neoplasia; the most common primary lung tumor in cats is an adenocarcinoma, and less common neoplasms include squamous cell carcinoma and anaplastic carcinoma.^11^, ^12^ Previous radiographic findings of pulmonary toxoplasmosis include diffuse interstitial to alveolar patterns and pleural effusion^2^, ^13^ Description of CT findings of pulmonary toxoplasmosis in cats are limited. One report describes a cat with multiple pulmonary nodules measuring up to a maximum of 15.7 mm diameter, resembling metastatic neoplasia.^1^ CT features of human patients with pulmonary toxoplasmosis include ground glass opacities, bilateral smooth septal and peribronchovascular thickening, atelectasis, random nodules, lymph node enlargement, and pleural effusion.^14^

The decision was taken to pursue surgical management in the first instance because of the extensive nature of this cat's disease. The imaging features and cytological assessment of the mass lesion raised concern that clindamycin alone would not penetrate all aspects of the suspected granuloma nor result in clinical resolution. Clinical and radiologic improvement occurs in human patients with *T gondii* mass lesions that are treated medically without surgical debulking.^15^, ^16^, ^17^ However, in the absence of other documented cases of solitary *T gondii* pulmonary mass‐like lesions in cats, medical treatment alone was not recommended. Adjuvant medical treatment was administered to increase the likelihood of clinical resolution, given that toxoplasmosis is usually a multisystemic disease. The authors acknowledge that even though apparent clinical resolution of this cat's *T gondii* granuloma was obtained, persistence of organisms in tissue cysts is likely, and clinical signs could recur in future in the event of immunosuppression.

## CONFLICT OF INTEREST DECLARATION

Authors declare no conflict of interest.

## OFF‐LABEL ANTIMICROBIAL DECLARATION

Clindamycin was used off‐label to treat toxoplasmosis.

## INSTITUTIONAL ANIMAL CARE AND USE COMMITTEE (IACUC) OR OTHER APPROVAL DECLARATION

Authors declare no IACUC or other approval was needed.

## HUMAN ETHICS APPROVAL DECLARATION

Authors declare human ethics approval was not needed for this study.
